# Nanoparticle-mediated magnetic hyperthermia is an effective method for killing the human-infective protozoan parasite *Leishmania mexicana in vitro*

**DOI:** 10.1038/s41598-018-37670-9

**Published:** 2019-01-31

**Authors:** Sarah L. Berry, Karen Walker, Clare Hoskins, Neil D. Telling, Helen P. Price

**Affiliations:** 1 Centre for Applied Entomology and Parasitology, School of Life Sciences, Keele University, Newcastle-under-Lyme, Staffordshire, ST5 5BG UK; 2 Central Electron Microscope Unit, School of Life Sciences, Keele University, Newcastle-under-Lyme, Staffordshire, ST5 5BG UK; 3 School of Pharmacy, Keele University, Newcastle-under-Lyme, Staffordshire, ST5 5BG UK; 4 Institute for Science and Technology in Medicine, Guy Hilton Research Centre, Keele University, Newcastle-under-Lyme, Staffordshire, ST4 7QB UK

## Abstract

Cutaneous leishmaniasis is a neglected tropical disease characterized by disfiguring skin lesions. Current chemotherapeutic options depend on toxic, expensive drugs that are both difficult to administer and becoming less effective due to increasing levels of resistance. In comparison, thermotherapy displays greater patient compliance and less adverse systemic effects, but there are still significant issues associated with this. The procedure is painful, requiring local anaesthetic, and is less effective against large lesions. Using nanoparticles to controllably generate heat in a localized manner may provide an alternative solution. Here we evaluate magnetic hyperthermia, using iron oxide magnetic nanoparticles, as a localized, heat-based method to kill the human-infective parasite *in vitro*. We assessed the effectiveness of this method against the differentiated, amastigote form of the parasite using three distinct viability assays: PrestoBlue, Live/Dead stain and a novel luciferase-based assay. Changes in amastigote morphology and ultrastructure were assessed by immunofluorescence, scanning and transmission electron microscopy. Our findings show that magnetic hyperthermia is an effective method to kill host-infective amastigotes, with morphological changes consistent with heat treatment. This method has the potential to be a step-change for research into new therapeutic options that moves away from the expensive chemotherapeutics currently dominating the research climate.

## Introduction

**Figure 1:**
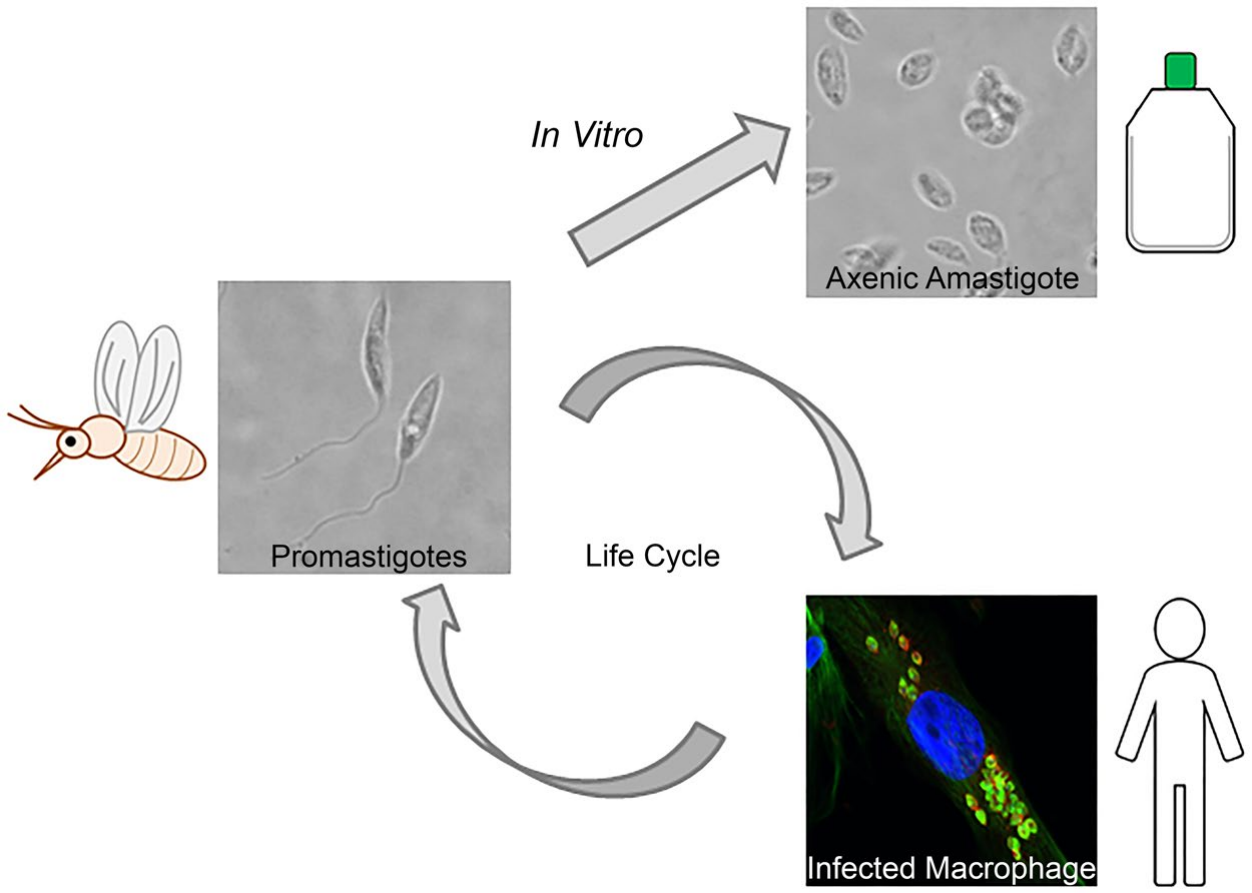
*Leishmania mexicana* life cycle stages grown *in vitro*. The *L. mexicana* parasite is present in its sandfly vector in the flagellated, promastigote form. The parasite is injected into humans following the bite of the infected sandfly. The parasite invades human mononuclear phagocytic cells, for example macrophages. Once inside the intracellular niche, the parasites transform into the amastigote form, which is rounder and has a shortened flagellum. For *Leishmania mexicana*, this amastigote form can be cultured axenically, *i.e*. in the absence of host mononuclear phagocytic cells. This allows the human infective stage of the parasite to be targeted initially in a simpler system, and parasite specific responses and phenotypes can be easily analyzed. Intracellular amastigotes were probed with anti α-tubulin antibody (green) and anti-HASPB (red) and the nucleus of the macrophage stained with DAPI (blue).

Leishmaniasis, a protozoan parasitic disease, causes significant mortality and morbidity globally^1^. The vector borne parasite that causes this disease does so by invading human mononuclear phagocytic cells, including macrophages (Fig. 1). The disease manifests in one of three main clinical forms that are dependent on the parasite species: visceral, cutaneous and mucocutaneous. Whilst visceral leishmaniasis is the most severe form, cutaneous leishmaniasis (CL) represents the majority of cases in humans^2^. CL is characterized by disfiguring skin lesions which often resolve without treatment. However, this process can take over a year, and leaves the individual at risk of secondary bacterial infections, scarring and parasite dissemination to other organs. The infected individual also acts as a reservoir for infection, resulting in parasite transmission. Current treatments depend largely on toxic drugs that are expensive and difficult to administer^3^. With resistant strains emerging to the current treatments^4^, and a recent sharp increase in the number of CL cases in the Middle East due to conflict and population displacement^5–7^, there is an urgent need for alternative therapeutics.

A viable alternative to the chemotherapeutic treatment of CL is the use of heat. Conventional thermotherapy uses a medical device to expose the surface of the skin lesion to a constant temperature (typically 50 °C), and has recently become the second line treatment in some geographical regions^8–10^. This method has been shown to have greater patient compliance and less adverse systemic effects than the current chemotherapeutic options^10,11^. However, the limited efficacy on large lesions, the painful procedure, the risk of cross contamination between patients and the need for large doses of localized anesthetic, means this method could be significantly improved^10,12^. One alternative is to use nanoparticles to controllably generate heat. The localized nature of this method is dictated by the presence of the nanoparticles: heat is only generated where the nanoparticles are present. Studies have been performed using nanoparticle-mediated photothermal therapy against leishmaniasis. These include the use of gold nanoparticles stimulated with microwave radiation^13^, and gold, silver, zinc, iron and titanium nanoparticles exposed to near infrared radiation^14^. One thermotherapy method that has not been explored for leishmaniasis is magnetic hyperthermia. This method uses magnetic nanoparticles (MNPs) to produce a localized heating effect when exposed to an alternating magnetic field, and has been reviewed recently^15^. This technology has been studied as a novel cancer treatment, where the MNPs can increase local tumor temperature to a critical level (~40–45 °C), inducing the death of malignant cells^16^. It has also shown promise when treating a cutaneous infection of *Staphylococcus aureus*, a major pathogen that often infects soft tissue wounds^17^. Magnetic hyperthermia has also been shown to kill *Crithidia fasciculata*, a non-pathogenic protozoan species^18^, but to our knowledge, no report of the use of this technology against human-infective protozoan species such as *Leishmania spp* has been shown. Harnessing heat generated by magnetic hyperthermia to target pathogens is therefore an attractive, non-chemotherapeutic and novel alternative approach to treating CL that could offer a convenient, cost effective solution to the issues associated with conventional thermotherapy.

The aim of this study was to assess whether magnetic hyperthermia has the potential to target the host-infective stage of this parasitic disease. We used *L. mexicana* axenic amastigotes in this work as they are the simplest system available, and allowed us to directly analyze the effect of magnetic hyperthermia on the amastigote. By targeting the human infective form, we show that magnetic hyperthermia kills the axenic amastigotes in a heat-dependent manner.

## Results and Discussion

**Figure 2:**
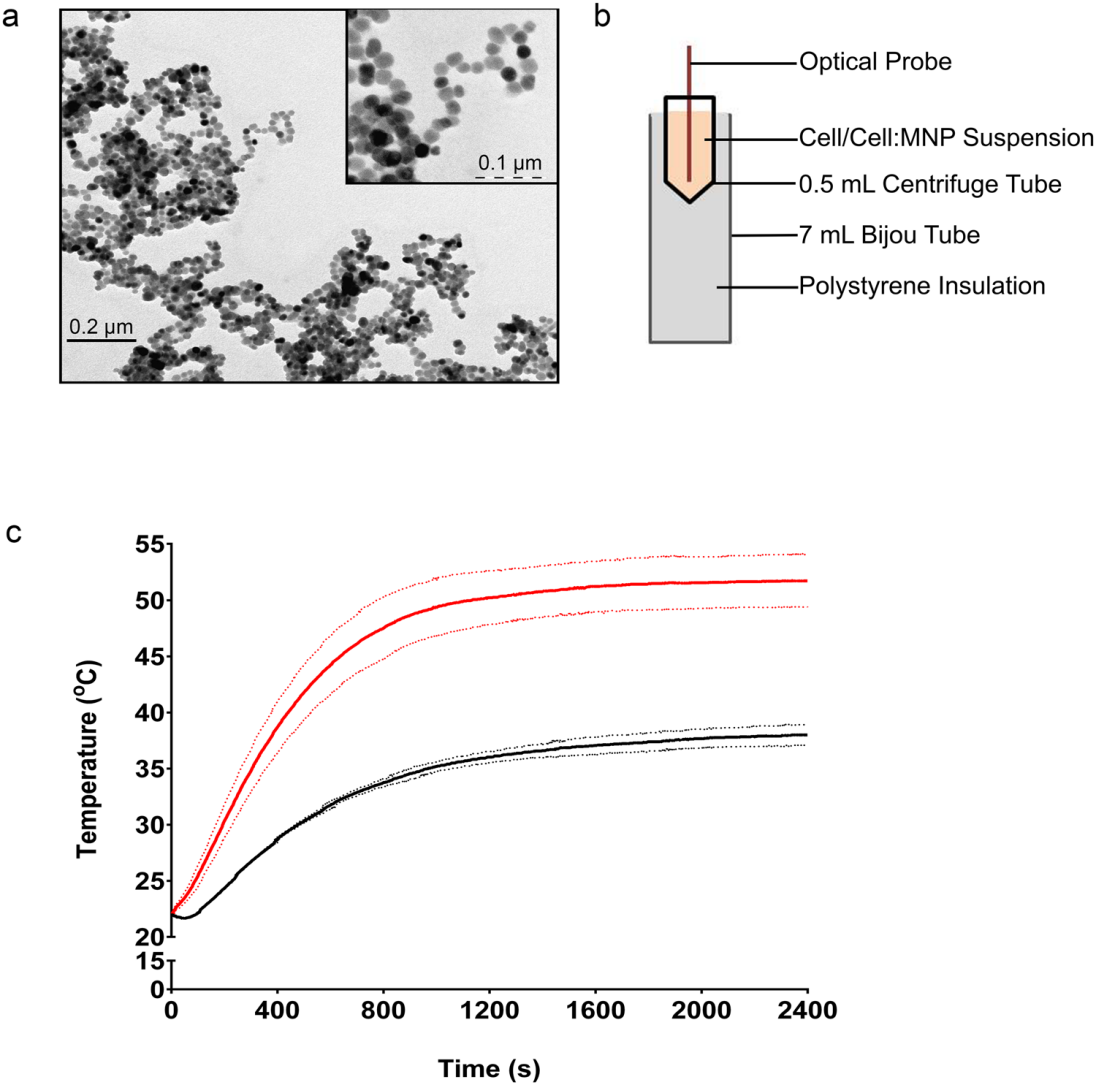
Characterizing the citric acid coated magnetic nanoparticles (MNPs). (**a**) Transmission electron micrograph of the MNPs. (**b**) Schematic of the sample holder used during magnetic hyperthermia. The optical probe is inserted through a hole in the centrifuge tube lid, and sealed with parafilm. The centrifuge tube is inserted into the bijou tube, which is filled with polystyrene for insulation. This also keeps the centrifuge tube in the center of the magnetic coil, for optimal heating. (**c**) The change in temperature of the axenic amastigote cell suspension during exposure to the alternating (AC) field in the presence (red) and absence (black) of MNPs. The graph depicts the average of three biological replicates (solid lines) ± standard deviation (dashed lines).

This work uses iron oxide MNPs to target axenic amastigotes *in vitro* (Fig. 1). Iron oxide nanoparticles have been used extensively in biomedical applications, with some particle types already approved by both the EU and FDA for use as either contrast agents or iron replacement therapies^19^. Iron oxide nanoparticles used in these settings are typically coated with hydrophilic ligands to provide stability in aqueous environments and improve biocompatibility. We initially coated magnetite MNPs with citric acid to produce stable, colloidal suspensions in water^20,21^. These MNPs have been characterized in previous studies^20,22^, but size, shape and stability were confirmed by dynamic light scattering (DLS) and transmission electron microscopy (TEM) (Table 1 and Fig. 2a). Prior to any cell based analysis, fetal bovine serum (FBS) was added to the colloidal MNP suspension (at a final concentration of 10%) and the solution was sonicated. This prevented the MNPs from precipitating out of solution when added to the cell media^23,24^. The FBS appears to coat the MNPs, nearly doubling their hydrodynamic radius (Table 1). This may correspond to a protein corona formed by serum proteins associating with the nanoparticle surface, which has been previously reported in the literature^22–24^.

**Table 1:** Properties of the citric acid coated MNPs, measured by dynamic light scattering.

Sample	Measurement	Mean (SD)
*Diluted in dH*_2_ *O*	Z-average (r.nm)	33.11 (3.54)
	Polydispersity Index	0.20 (0.05)
	Zeta Potential (mV)	−51.70 (11.59)
*Sonicated with FBS, diluted with dH*_2_ *O*	Z-average (r.nm)	59.76 (1.80)
*Sonicated with FBS, diluted with complete Schneider’s media (pH 5.5)*	Z-average (r.nm)	55.80 (3.39)

**Figure 3:**
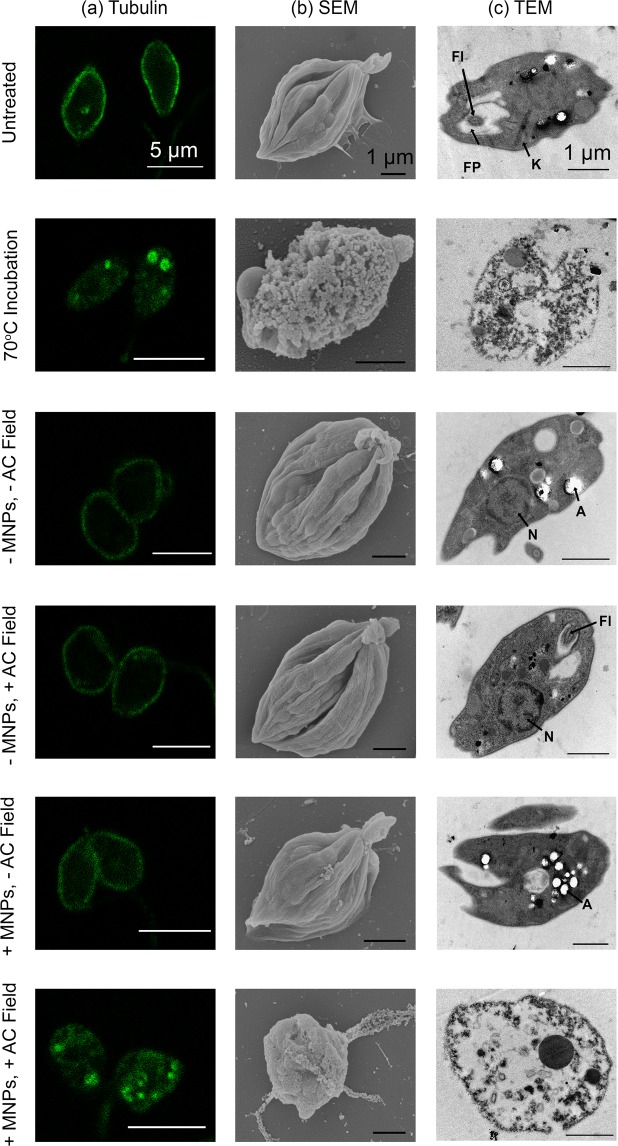
Microscopic analysis of cellular and ultrastructural alterations following treatment. (**a**) Immunofluorescence of axenic amastigotes probed with anti-α-tubulin (green). (**b**) Scanning electron micrographs of axenic amastigotes. (**c**) Transmission electron micrographs of axenic amastigotes. FP; flagellar pocket, Fl; flagellum, K; kinetoplast, N; nucleus, A; acidocalcisome. Positive (70 °C treated) and negative (untreated) controls are displayed. Representative images are depicted.

The MNPs do not appear to be internalized by the axenic amastigotes, which was determined by measuring the intracellular iron content (see Supplementary Table S1). This correlates with the flow cytometry data (see Supplementary Fig. S1); specifically there is no change in the intensity of the side scatter of the axenic amastigotes in the presence of the MNPs, indicating no MNP uptake^25^. TEM further corroborated this data, where no evidence of MNP uptake could be identified (Fig. 3). This data was not altogether unexpected because, whilst the MNPs (with a hydrodynamic diameter of ~66 nm; Table 1) are approximately 50 times smaller than the size of the axenic amastigotes (3–5 µm in diameter), the amastigotes only take up macromolecules from the extracellular environment via a small, localized region on the cell membrane known as the flagellar pocket^26^. The MNPs therefore remain in the medium following incubation with the parasites, and any heat generated will occur in the space surrounding the cells. This creates a microenvironment similar to that within the phagolysosome of an infected macrophage. We then proceeded to assess whether the presence of MNPs within the medium would generate sufficient heat to affect the parasites directly, using the configuration shown in Fig. 2b. A non-specific heating effect was observed in our device due to the power dissipated in the coils used to generate the AC magnetic field (Fig. 2c). The temperature this reached is ~37 °C, which is ~20 °C lower than the solution temperature reached in the presence of the MNPs. Whilst this baseline of 37 °C is slightly higher than the 32 °C that the *L. mexicana* axenic amastigotes are typically cultured at, by maintaining this temperature any potential artefact associated with incubation at lower temperatures was avoided. In order to ensure there were no ultrastructural changes, the axenic amastigotes were submitted to analysis by microscopy (Fig. 3). In all microscopic analyses, no difference was observed between the untreated control cells, and the cells exposed to the magnetic field in the absence of MNPs (−MNP, +AC Field, Fig. 3).

Immunofluorescence microscopy was used to visualize α-tubulin in the axenic amastigotes. *Leishmania spp* have a tubulin-based cytoskeleton that consists largely of a densely packed network of sub-pellicular microtubules. Our results indicate a regular distribution of α-tubulin, consistent with the cytoskeleton in in all samples, with the exception of the control cells (incubated at 70 °C), and the cells treated with magnetic hyperthermia (+MNP, +AC field; Fig. 3a). Discrete foci of tubulin are observed in these two samples instead of the regular cytoskeletal distribution. This indicates that exposure to heat affects the distribution or integrity of the microtubular network within the axenic amastigotes. Disruption of the microtubular network following magnetic hyperthermia has also been seen previously in HeLa cells (a cervical cancer cell line), and was attributed to extracellular heating^27^. This indicates a fundamental mechanism is involved in heat response, and explains the similarities in phenotype between the control cells incubated at 70 °C and the sample subjected to magnetic hyperthermia.

Samples were then analyzed by scanning electron microscopy (SEM) and TEM to investigate changes to the cellular ultrastructure following treatment (Fig. 3b,c respectively). In agreement with the immunofluorescence microscopy, SEM analysis shows normal morphology in all samples with the exception of the control cells (incubated at 70 °C), and the cells treated with magnetic hyperthermia (+MNP, +AC field; Fig. 3). Despite the temperature difference in the two treatments, the cells that were treated with magnetic hyperthermia display a grossly abnormal cell surface that is similar to the control cells (incubated at 70 °C). This shows that magnetic hyperthermia is as effective as direct heat treatment with regards to ultrastructure disruption. Further analysis of our samples by TEM showed a striking phenotype in the control cells (incubated at 70 °C) and the cells treated with magnetic hyperthermia (+MNP, +AC field; Fig. 3c). Complete destruction of the internal cellular architecture is seen in cells from both samples. This indicates that the dramatic changes observed in the amastigotes following magnetic hyperthermia are consistent with heat-induced cell damage and death.

**Figure 4:**
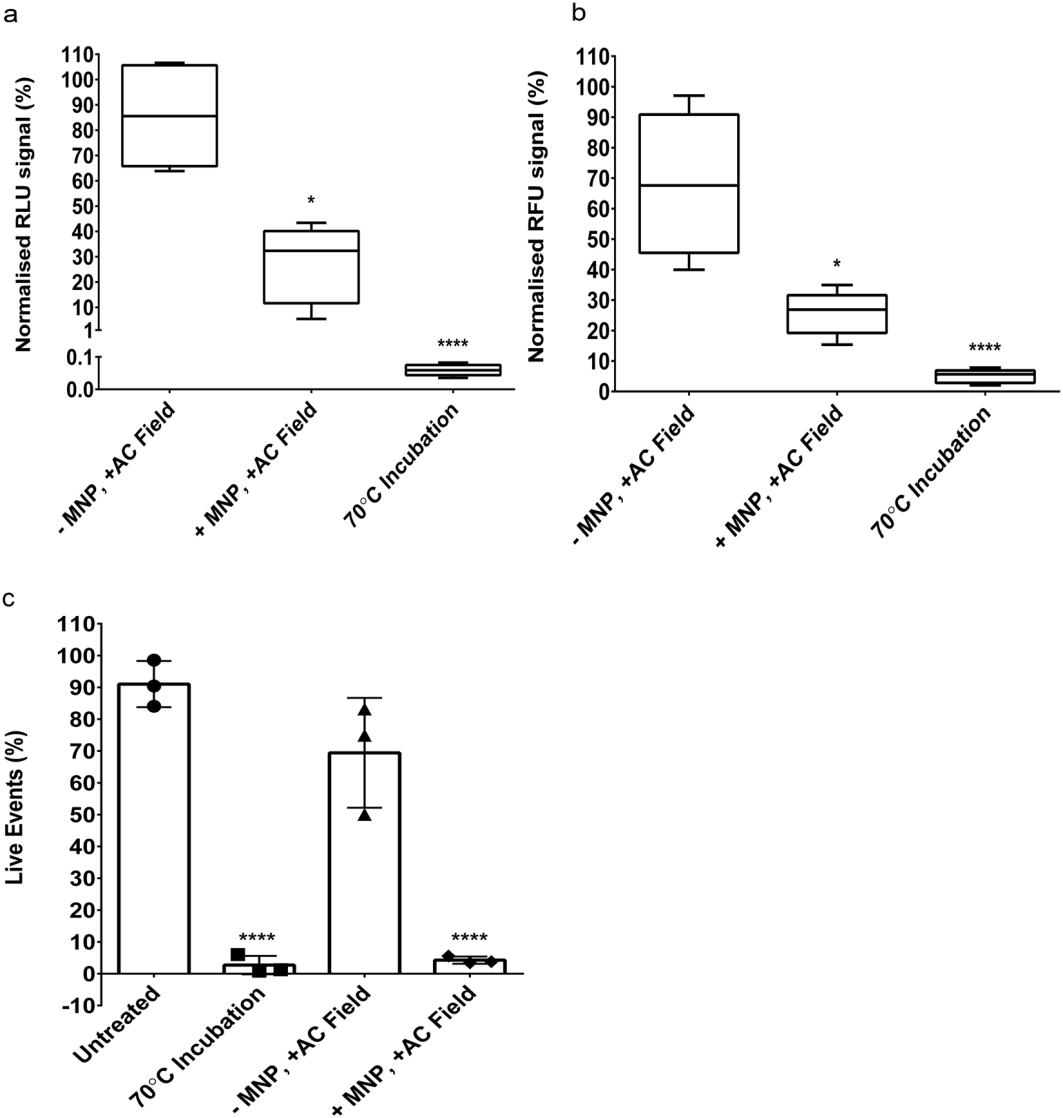
Viability of the axenic amastigotes following treatment. (**a**) Luciferase-based viability assay of the axenic amastigotes following treatment. Values depicting percentage change in luminescence relative to the untreated control (not exposed to the AC field) are shown as box and whisker plots (n = 12 from four independent experiments). (**b**) Resazurin-based viability assay of the axenic amastigotes following treatment. Mean values depicting percentage change in fluorescence relative to the untreated control (not exposed to the AC field) are shown as box and whisker plots (n = 12 from four independent experiments). (**c**) Summary of the number of gated, live axenic amastigotes following treatment. Mean values are shown (n = 3) ± SEM. Full data sets are shown in Supplementary Fig. S2.

Axenic amastigote viability was then assessed using three distinct methods: a luciferase-based luminescent method^28^, a resazurin-based fluorescent method^29^ and a fluorescent live/dead amine reactive dye for flow cytometry^30^. All assays were performed on the NanoLuc-PEST luciferase-expressing *L. mexicana* cell line, which is referred to as L. mx NanoLucP^28^. The presence of the iron nanoparticles alone (0.35 µM per sample) had no effect on amastigote viability. This corroborates work published by Vale-Costa *et al*., who showed that incubating parasites with less than 0.56 mM iron had no effect on viability^31^. However, the viability of the axenic amastigotes was significantly reduced by ~70%, following magnetic hyperthermia treatment (Fig. 4a,b; p = 0.016). This was further confirmed using the live/dead flow cytometric method (Fig. 4c, Supplementary Fig. S2), which shows a decrease of 96% in the number of ‘live’ cells (p < 0.0001) following magnetic hyperthermia.

This work is the first report showing that magnetic hyperthermia can efficiently kill the causative agent of cutaneous leishmaniasis, and provides insight into a potential fundamental mechanism involved in the cellular response of the amastigotes to heat. Importantly, this study lays the groundwork for the exploration of magnetic hyperthermia to target amastigotes in their intracellular niche. The future direction of this project will involve specifically targeting the amastigote within its intracellular niche. This will involve the use of both macrophage infection models and animal models. The data presented here indicates the potential for re-purposing this important emerging nanotechnology as a powerful alternative treatment option for CL, a disfiguring neglected tropical disease.

## Methods

### Parasite culture

A transgenic, luciferase expressing cell line of the *L. mexicana* strain MNYC/BZ/62/M379 was maintained *in vitro* in the procyclic promastigote stage in Schneider’s media pH 7.0 supplemented with 10% (v/v) FBS, 100 U/mL penicillin, 100 μg/mL streptomycin and 40 µg/mL geneticin. This cell line constitutively expresses luciferase from deep sea shrimp that is attached to a PEST degradation sequence^28,32^. Axenic amastigote differentiation was performed as described previously^33^, in Schneider’s media pH 5.5 supplemented with 10% (v/v) FBS, 100 U/mL penicillin, 100 μg/mL streptomycin and 40 µg/mL geneticin.

### Magnetic MNP Production

Commercially available magnetite (Fe_3_O_4_) nanopowder was obtained from Sigma (637106, lot MKBD8421V and MKBG0737V). Stable, colloidal suspensions were produced using a procedure similar to that described elsewhere^20,21^. Briefly, 20 mg/mL of nanopowder was re-suspended in deionized H_2_O, and the pH subsequently decreased to ~2 using concentrated hydrochloric acid. The suspension was sonicated for 2 minutes, powdered citric acid (15 mg/mL) was added and the pH subsequently increased to ~5 using concentrated sodium hydroxide. The solution was heated at 80 °C for 30 minutes, interspersed with 2 minute sonication every 10 minutes. Excess citric acid was then removed by magnetic decantation, and washed twice with acetone. MNPs were re-suspended in deionized H_2_O (pH > 9), and subjected to sonication, vortexing, centrifugation and magnetic decantation to remove large clusters, leaving a stable magnetic MNP suspension. This was filter-sterilized using a 0.2 µm filter.

### Iron Quantification

The concentration of the colloidal nanoparticle suspension was quantified using the FerroZine assay^31,34^. Briefly, 100 µL 6 M HCl was incubated with the MNP-containing sample (200 µL) for 60 minutes at 65 °C. An aliquot of the digested sample (150 µL) was incubated with equal amounts of 6.6 M hydroxylamine hydrochloride for 2.5 hours at room temperature. An aliquot of the reduced sample (100 µL) was treated with 700 µL of 2 mM FerroZine in 50 mM HEPES buffer, and incubated for 60 minutes at room temperature in the dark. Absorbance was read at 562 nm on the Promega GloMax detection system. Standard curves were plotted using iron standards.

The intracellular iron concentration was quantified using inductively coupled plasma atomic emission spectroscopy (ICP-AES). Axenic amastigotes were seeded into T25 flasks (2 × 10^8^ cells/flask). FBS-coated MNPs (80 µg/mL) were incubated with cells for 1, 4 and 24 hours. ‘0 hr’ controls were treated with an equivalent volume of deionized H_2_O + FBS for 24 hours. Cells were washed four times in PBS and re-suspended at a concentration of 1 × 10^7^ cells/mL in 70% nitric acid and stored at 4 °C prior to analysis. Samples (1 mL) were diluted in 10 mL deionized H_2_O prior to ICP-AES. A calibration curve was produced using iron standards at the following concentrations: 10, 5, 1, 0.5, 0.1, 0.05 mg/L. The iron (Fe) content was determined at 259.94 nm and calculated per cell (pg cell^−1^).

### Magnetic Hyperthermia

Aliquots of cell suspension (2.5 × 10^8^ axenic amastigotes/mL) in the presence or absence MNPs (0.2 mg/mL) were submitted to alternating magnetic fields using a NanoHeat magnetic hyperthermia fluid device (NanoScience Laboratories). A 2x solution of MNPs was sonicated with 10% (v/v) FBS for 10 minutes in 30 second pulses using the Biorupter UCD 200 Diagenode sonicator. Equivalent water: FBS samples were used for controls. Samples (400 µL) were placed in a 0.5 mL centrifuge tube, in a poly(styrene)-insulated 7 mL bijou tube at the center of a temperature regulated coil (Fig. 2b). Water, connected to the mains water supply (~22 °C), was circulated through the coil. This helped to keep the temperature of the target solution at ~37 °C, preventing any potential artifacts due to a decrease in temperature. Cells were exposed to a high frequency (f = 452 kHz) alternating magnetic field of 30 mT for 40 minutes. The sample temperature was recorded using an optical probe. Following hyperthermia, cells were incubated at 32 °C for 20 hours.

### Cell Viability Assays

For each assay, a positive control (consisting of axenic amastigotes incubated at 70 °C for 5 minutes) was included. Incubation at this temperature ensured complete loss of cell viability.

PrestoBlue-based viability assays were performed on L. mxNanoLucP axenic amastigotes in a 96 well plate format following treatment and incubation. This method measures mitochondrial activity of the axenic amastigote^35^. Cells were diluted to a final concentration of 2 × 10^6^ cells. PrestoBlue was added immediately (1:10 dilution). Plates were incubated in the dark at 32 °C for 4.5 hours. Fluorescence was measured using the Promega GloMax detection system, using the green optical kit (ex/em = 525/580–640 nm).

Luciferase-based viability assays were performed on L. mxNanoLucP axenic amastigotes in a 96 well plate format following treatment and incubation. The NanoLuc-PEST luciferase (expressed in the transgenic *L. mexicana* cell line) is a parasite-specific viability marker that measures proteasome turnover^28,32^. Cells were diluted to a final concentration of 5 × 10^5^ per well. Detection of luciferase activity was performed using the Nano-Glo assay kit (Promega), as described by the manufacturer. Bioluminescence was measured using the Promega GloMax detection system.

Flow cytometry-based viability assays were performed on L. mxNanoLucP axenic amastigotes using a commercial live/dead fixable staining kit (Invitrogen, L34971). This method has the added advantage of allowing internalization of MNPs by the amastigotes to be assessed, alongside viability. Briefly, 4 × 10^7^ cells were centrifuged, washed once in PBS and re-suspended in 1 mL PBS. Live/Dead viability stain was added at a concentration of 1 µL per 1 × 10^7^ cells, and incubated at room temperature for 30 minutes. All steps from this point on were performed in the dark, to prevent bleaching. Cells were washed once in PBS, re-suspended in 1 mL of 4% formaldehyde: PBS, and incubated at room temperature for 15 minutes. Cells were washed once in PBS, and then re-suspended in 1 mL PBS. Samples were diluted to 1 × 10^7^ cells/mL prior to analysis. Cell analysis was performed using the Guava EasyCyte 6-2L flow cytometer, and InCyte software (Merck Millipore). The flow cytometer was calibrated before each use, as described in the manufacturer’s protocol. 20, 000 events were counted per sample. The 488 nm laser line was used to stimulate the fluorophore, whilst the Yel-B (583/26) emission filter was used for detection. Sample gating was performed initially against size and intracellular composition (FSC/SSC respectively) of the axenic amastigotes. Live/dead gating was then performed using the untreated, stained control (which represents the fraction of viable cells within the population).

### Statistical analysis

Data for fluorescence and luminescence viability assays were analyzed using the same method. Data was transformed by taking the percentage decrease in signal (either RLU or RFU for luminescence and fluorescence respectively) for samples in the absence (-MNPs) or presence (+MNPs) of MNPs when exposed to the AC field, compared to paired samples not exposed to the AC field:

$$Normalised\,signal\,( \% )=100-((\frac{no\,field-field}{no\,field})\ast 100)$$

This data was then tested for normality (using the Shapiro-Wilk normality test; see Supplementary Table S2) and homogeneity of variance (using the Levene’s test; see Supplementary Table S3). The fluorescence and luminescence data violated these assumptions. Statistical significance was therefore assessed using the Kruskal-Wallis non-parametric test, and comparisons performed using Dunn’s post-hoc test. These tests have a significance level (or alpha) of 0.05.

The percentage data obtained from the flow cytometric method was transformed using arcsin prior to analysis. This was tested for normality and homogeneity as described above (see Supplementary Tables S2 and S3), and didn’t violate either of these assumptions. An Ordinary One-Way ANOVA with the Tukey’s post-hoc test was used. This test has a significance level (or alpha) of 0.05.

All statistical analyses were performed in IBM SPSS Statistics 24.

### Microscopy

Samples were prepared for immunofluorescent microscopy using 1 × 10^7^ cells per sample. L. mxNanoLucP axenic amastigotes were fixed in 4% formaldehyde: PBS then washed twice in PBS. Samples were incubated with 0.1% Triton X-100/PBS for 10 minutes at room temperature, and blocked with Image iT for 30 minutes at room temperature. Cells were probed with anti-α-tubulin antibody TAT1 (diluted in PBS; provided by Professor Keith Gull, University of Oxford) for 60 minutes at room temperature, then washed three times in PBS. Cells were incubated with Alexa Fluor anti-mouse 488 (diluted in PBS) in the dark for 60 minutes at room temperature, and then washed three times in PBS. Cells were re-suspended in 100 µL PBS and adhered to poly-L-lysine slides for 30 minutes at room temperature. Coverslips were mounted using Prolong mounting media and set overnight at room temperature. Slides were kept at 4 °C prior to visualization using the Zeiss LSM 710 microscope. Microscope settings are detailed in Supplementary Table S4.

Samples were prepared for scanning electron microscopy using 2 × 10^7^ cells per sample. L. mxNanoLucP axenic amastigotes were washed three times in serum-free Schneider’s media (pH 5.5) and once in PBS. Samples were seeded onto poly-L-lysine coated 12 mm coverslips, washed once in PBS, then washed once in sodium cacodylate buffer (0.1 M sodium cacodylate/2 mM CaCl2 (pH 7.4)). Samples were fixed in 2.5% glutaraldehyde: sodium cacodylate buffer for 2 hours, washed three times in sodium cacodylate buffer, then post-fixed in 1% osmium tetroxide: sodium cacodylate buffer for 1 hour. Samples were washed six times in sodium cacodylate buffer (5 minutes per wash), then incubated in a saturated aqueous solution of sodium thiocarbohydrazide for 20 minutes. Samples were washed six times in distilled water (5 minutes per wash), then incubated in 1% osmium tetroxide: sodium cacodylate buffer for 2 hours. Samples were washed six times in distilled water, and the previous four steps were repeated. Samples were dehydrated through an increasing ethanolic series (70%, 80%, 90%, 100%, and 100% dry ethanol), critical point dried from liquid carbon dioxide, and then mounted on scanning electron microscopy stubs using double sided carbon adhesive pads. Samples were visualized using a Hitachi S-4500 field emission scanning electron microscope at an accelerating voltage of 5 kV. Micrographs were captured using Quartz PCI version 9 capture system (Quartz Imaging Corporation, Vancouver, Canada).

Samples were prepared for transmission electron microscopy using 2 × 10^7^ cells per sample. L. mxNanoLucP axenic amastigotes were washed three times in serum free Schneider’s media (pH 5.5) and once in PBS. Samples were seeded onto poly-L-lysine coated Aclar film, (Agar Scientific, Stansted, UK), washed once in PBS, then washed once in sodium cacodylate buffer (0.1 M sodium cacodylate/2 mM CaCl_2_ (pH 7.4)). Samples were fixed in 2.5% glutaraldehyde: sodium cacodylate buffer for two hours, washed three times in sodium cacodylate buffer, then post-fixed in 1% osmium tetroxide: sodium cacodylate buffer for 1 hour. Samples were washed three times in sodium cacodylate buffer (5 minutes per wash), then dehydrated through an increasing ethanolic series (70%, 80%, 90%, 100%, and 100% dry ethanol), then infiltrated and embedded in Spurr resin (Agar Scientific, Stansted, UK). Ultrathin sections (~100 nm thick) were cut using a Reichert Ultracut E microtome on to 200 mesh thin bar copper grids (G2002; Agar Scientific, Stansted, UK), stained with 2% ethanolic uranyl acetate and 2% aqueous lead citrate and visualized using a JEOL 1230 transmission electron microscope operated at an accelerating voltage of 100 kV. Micrographs were captured using a Megaview III digital camera and analysSIS® software (Olympus Soft Imaging Systems GMBH, Germany).

## Supplementary information

Supplementary Information

## Data Availability

Any datasets generated during and/or analysed during the current study that are not included in the supplementary information are available from the corresponding author on reasonable request.
