# Supplementary material for: Nanoparticle-mediated magnetic hyperthermia is an effective method for killing the human-infective protozoan parasite Leishmania mexicana in vitro
Source: Sci Rep. 2019 Jan 31;9:1059. doi: 10.1038/s41598-018-37670-9 (PMC6355871; doi:10.1038/s41598-018-37670-9)

**Nanoparticle-mediated magnetic hyperthermia is an effective method for killing the human-infective protozoan parasite *Leishmania mexicana* *in vitro*.**

Sarah L Berry ^1^, Karen Walker ^2^, Clare Hoskins ^3^, Neil D Telling ^4*^ and Helen P Price^1*^

^1.^ Centre for Applied Entomology and Parasitology, School of Life Sciences, Keele University, ST5 5BG, UK.

^2.^ Central Electron Microscope Unit, School of Life Sciences, Keele University, Newcastle-under-Lyme, Staffordshire, ST5 5BG, UK.

^3.^ School of Pharmacy, Keele University, Newcastle-under-Lyme, Staffordshire, ST5 5BG, UK.

^4.^ Institute for Science and Technology in Medicine, Guy Hilton Research Centre, Keele University, Newcastle-under-Lyme, Staffordshire, ST4 7QB, UK.

*Corresponding author

Email:

HPP: [h.price@keele.ac.uk](mailto:h.price@keele.ac.uk)

NDT: [n.d.telling@keele.ac.uk](mailto:n.d.telling@keele.ac.uk)

# Supplementary Information

**Supplementary Table S1 - Intracellular iron content of *L. mexicana* axenic amastigotes following incubation with FBS-coated MNPs.**

|  | **Average Fe content/cell (pg)** | **s.d.** | ***p* value** |
| --- | --- | --- | --- |
| *0 hr* | 0.023 | 0.010 | N/A |
| *1 hr* | 0.024 | 0.004 | 0.99 |
| *4 hr* | 0.026 | 0.002 | 0.77 |
| *24 hr* | 0.035 | 0.005 | 0.05 |

Intracellular iron content after 1, 4 and 24 hours was directly compared to the 0 hour control. Statistical analysis was performed in IBM SPSS Statistics 24 using a one-way ANOVA with the Games-Howell post-hoc test (n=6 from two biological replicates). This test has a significance level (or alpha) of 0.05.

**Supplementary Table S2 – Statistical analysis for normality.**

|  | Conditions | Shapiro-Wilk Test | | |
| --- | --- | --- | --- | --- |
|  |  | Test statistic | Degrees of freedom | p value |
| Luminescence-Based Viability Assay | -MNP, +AC Field | 0.691 | 12 | 0.001 |
|  | +MNP, +AC Field | 0.802 | 12 | 0.010 |
|  | 70°C Incubation | 0.933 | 12 | 0.417 |
| Fluorescence-Based Viability Assay | -MNP, +AC Field | 0.837 | 12 | 0.025 |
|  | +MNP, +AC Field | 0.934 | 12 | 0.425 |
|  | 70°C Incubation | 0.892 | 12 | 0.125 |
| Flow Cytometric Viability Assay | -MNP, +AC Field | 0.940 | 3 | 0.528 |
|  | +MNP, +AC Field | 0.888 | 3 | 0.348 |
|  | 70°C Incubation | 0.853 | 3 | 0.249 |
| Intracellular Iron Content | 0 hours | 0.772 | 6 | 0.033 |
|  | 1 hour | 0.908 | 6 | 0.423 |
|  | 4 hours | 0.961 | 6 | 0.828 |
|  | 24 hours | 0.846 | 6 | 0.146 |

**Supplementary Table S3 – Statistical analysis for homogeneity of variance.**

|  | Homogeneity of Variance (Levene’s Test) | | | |
| --- | --- | --- | --- | --- |
|  | Test statistic (F) | df1 | df2 | p value |
| Luminescence-Based Viability Assay | 50.716 | 2 | 33 | <0.001 |
| Fluorescence-Based Viability Assay | 77.545 | 2 | 33 | <0.001 |
| Flow Cytometric Viability Assay | 4.403 | 2 | 6 | 0.067 |
| Intracellular Iron Content | 43.207 | 3 | 20 | <0.001 |

**Supplementary Table S4 – Zeiss LSM 710 microscopy settings.**

| Objective Lens | 100x 1.45 NA oil |
| --- | --- |
| Bit depth | 16 bit |
| AF488 laser | 5% intensity |
| AF488 pinhole | 78 (1 AU) |
| Detector | 490-579 nm |
| Gain | 995 V |
| Offset | 0 |
| Digital gain | 0.3 |

**Supplementary Figure S1. Flow cytometric analysis of cell granularity, to assess MNP uptake by axenic amastigotes.**

The three columns indicate results for three biological replicates. Histograms depict side scatter (SSC) against number of gated cells.

**

**

**Supplementary Figure S2. Live/dead flow cytometric analysis following treatment, for all biological replicates.**

The three columns indicate results for the three biological replicates. Graphs depict live/dead staining of the axenic amastigotes. Viability was assessed using the amine reactive live/dead fixable stain (FSC vs YEL-B). Axes are depicted as a log scale.


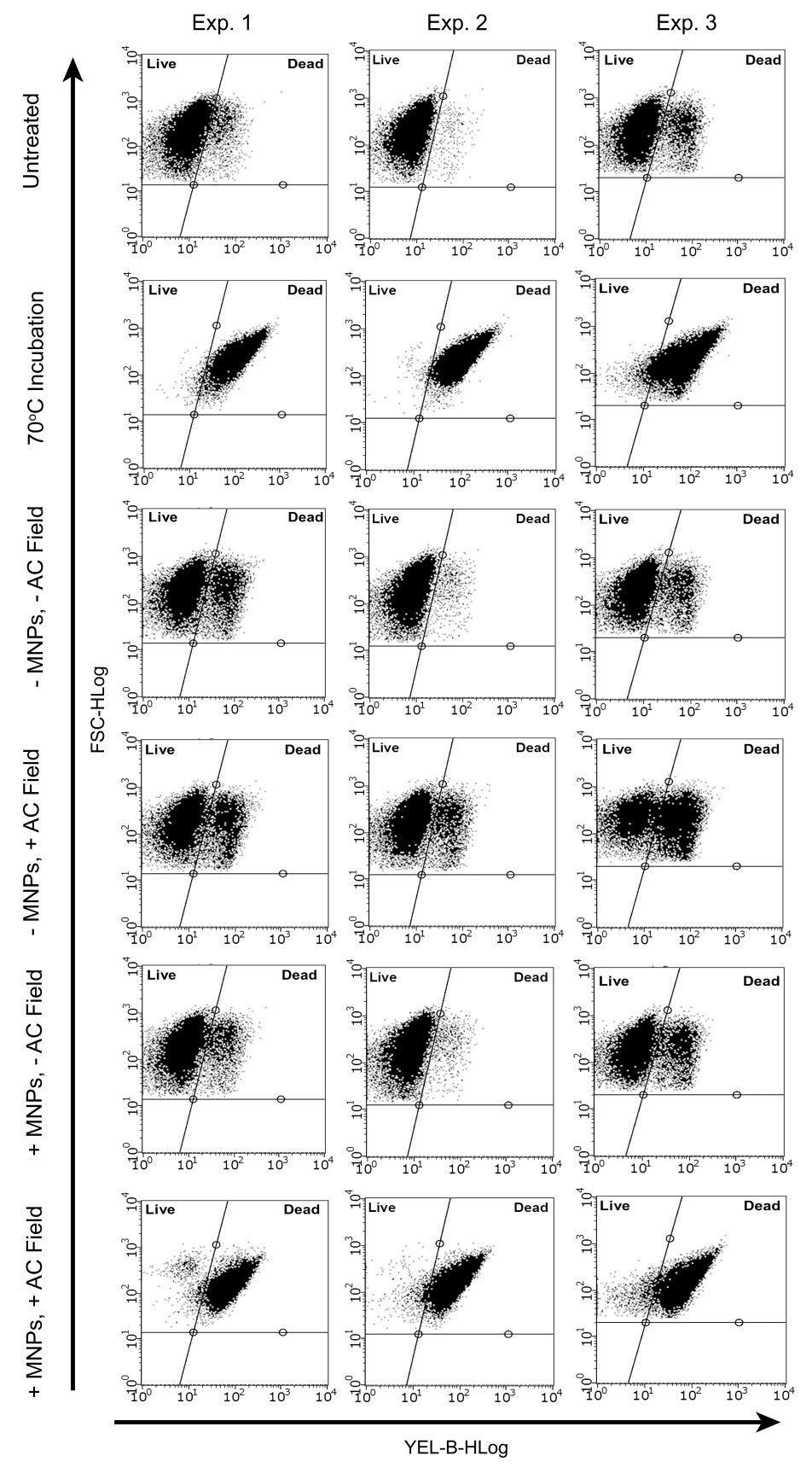

Supplement: Supplementary file 1 — Supplementary Information [file 41598_2018_37670_MOESM1_ESM.docx]
